# Inferring the Source of Transmission with Phylogenetic Data

**DOI:** 10.1371/journal.pcbi.1003397

**Published:** 2013-12-19

**Authors:** Erik M. Volz, Simon D. W. Frost

**Affiliations:** 1 Department of Infectious Disease Epidemiology, Imperial College London, London, United Kingdom; 2 Department of Veterinary Medicine, University of Cambridge, Cambridge, United Kingdom; University of California San Diego, United States of America

## Abstract

Identifying the source of transmission using pathogen genetic data is complicated by numerous biological, immunological, and behavioral factors. A large source of error arises when there is incomplete or sparse sampling of cases. Unsampled cases may act as either a common source of infection or as an intermediary in a transmission chain for hosts infected with genetically similar pathogens. It is difficult to quantify the probability of common source or intermediate transmission events, which has made it difficult to develop statistical tests to either confirm or deny putative transmission pairs with genetic data. We present a method to incorporate additional information about an infectious disease epidemic, such as incidence and prevalence of infection over time, to inform estimates of the probability that one sampled host is the direct source of infection of another host in a pathogen gene genealogy. These methods enable forensic applications, such as source-case attribution, for infectious disease epidemics with incomplete sampling, which is usually the case for high-morbidity community-acquired pathogens like HIV, Influenza and Dengue virus. These methods also enable epidemiological applications such as the identification of factors that increase the risk of transmission. We demonstrate these methods in the context of the HIV epidemic in Detroit, Michigan, and we evaluate the suitability of current sequence databases for forensic and epidemiological investigations. We find that currently available sequences collected for drug resistance testing of HIV are unlikely to be useful in most forensic investigations, but are useful for identifying transmission risk factors.

## Author Summary

Molecular data from pathogens may be useful for identifying the source of infection and identifying pairs of individuals such that one host transmitted to the other. Inference of who acquired infection from whom is confounded by incomplete sampling, and given genetic data only, it is not possible to infer the direction of transmission in a transmission pair. Given additional information about an infectious disease epidemic, such as incidence of infection over time, and the proportion of hosts sampled, it is possible to correct for biases stemming from incomplete sampling of the infected host population. It may even be possible to infer the direction of transmission within a transmission pair if additional clinical, behavioral, and demographic covariates of the infected hosts are available. We consider the problem of identifying the source of infection using HIV sequence data collected for clinical purposes. We find that it is rarely possible to infer transmission pairs with high credibility, but such data may nevertheless be useful for epidemiological investigations and identifying risk factors for transmission.

This is a *PLOS Computational Biology* Methods article.

## Introduction

Phylogenetic trees reconstructed from sequences of pathogens contain information on the past transmission dynamics that would be difficult, if not impossible, to obtain through other means. Over the past two decades, a number of approaches have been proposed to extract epidemiologically relevant information from viral phylogenies, particularly from highly variable RNA viruses such as HIV-1, hepatitis C virus, and influenza A virus [1]. With the advent of high-throughput sequencing, these approaches can also be applied to help understand bacterial spread [2].

Although many studies have focused on the ‘phylodynamics’ [3], [4] of infectious disease transmission at the population level, there have been a number of studies that have focused more on the ability of molecular sequence data to inform transmission at the level of pairs or small groups of individuals. Molecular epidemiological analysis of couples with discordant HIV status have demonstrated that infection of the initially uninfected partner may often be from a third party [5]. Sequence data have also been used in a forensic setting [6], [7], most famously in the Florida dentist case [8]. Identifying the source of infection from genetic data is known to be confounded by many sources of error. The similarity of pathogen sequence data collected from a transmission pair depends, among other factors, on the time since transmission, immunological pressure on the pathogen, the substitution rate of the pathogen within host, and how the substitution rate changes over time within hosts. Provided a realistic model of how pathogen sequences diverge over time, it is possible to calculate the probability that the consensus sequence in a recipient of infection diverged in a given span of time from a putative source of infection [9]–[11].

Recently, there has been rapid development of methods to identify transmission sources under the assumption of complete sampling, i.e. under the assumption that every infected individual is represented in the phylogeny. These methods have yielded many valuable insights into the spread of nosocomial infections [12], Mycobacterium tuberculosis [13], foot-and-mouth disease virus, and avian influenza between farms [14]. Nevertheless, for many human pathogens, incomplete sampling is the rule. In the case of HIV, sequencing of the *pol* gene is now routine in many countries for surveillance of drug resistance, but even so, sample coverage is far from complete.

**Figure 1:**
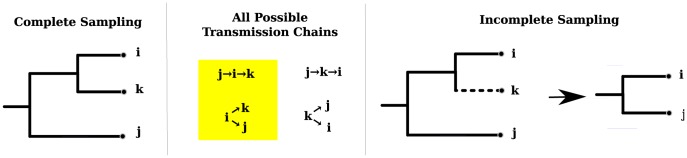
Four transmission trees between hosts *i*, *j* and *k* are shown (center) that are consistent with the pathogen gene genealogy (left). If the host *k* is not sampled, the resulting gene genealogy is shown at right. Transmission trees where *i* directly interacts with *j* are highlighted. The unsampled unit *k* may act as either a common source of infection for *i* and *j* or a an intermediate infection between *i* and *j*.

Figure 1 illustrates errors that can be introduced by incomplete sampling. For example, it is possible that two hosts with genetically similar virus were both infected by a common source who was not sampled. Therefore, calculation of the probability that *i* infected *j* should account for the possibility that an unobserved individual *k* infected both *i* and *j* (second panel). Similarly, it is possible that *i* infected an unsampled individual *k* who went on to infect *j*. Due to the uncertainty stemming from incomplete sampling, viral sequence data have often been used as a test to disconfirm a putative transmission pair. For example, in the context of HIV, a phylogeny estimated from a pair of sequences in a putative transmission pair, along with a set of sequences from a suitable background population (e.g. infected individuals with a matching geographic and risk-behavior profile), can be used to detect if the putative donor is relatively distant in evolutionary terms from the recipient [15]. If the putative donor is not monophyletic with the recipient, it is less likely that the putative donor is the true source of infection. However, even if the donor and recipient sequences are not monophyletic, there are scenarios where the putative transmission pair is genuine. For example, it is possible that the putative donor is a common source of infection for all sampled cases in the donor-recipient clade. This is illustrated in Figure 1, in which donor *i* infects both *j* and *k*, yielding a polyphylous relationship between *i* and *j*. As it is impossible to rule out the possibility that an unsampled individual or unobserved chain of transmissions connects a putative donor and recipient, it has been impossible to properly define the statistical power of tests for confirming or disconfirming transmission pairs from phylogenetic data.

Due to the problems involved in incomplete sampling, relatively little work has been performed to identify potential sources of infection - i.e. understanding transmission at an individual level - using population-level datasets collected for clinical or surveillance purposes. A notable exception is a study of HIV-positive men who have sex with men (MSM) in Brighton, UK [16], which, through a combination of diagnosis times and sequence data, attempted to identify the source of transmission for 159 cases of recent HIV infection. A single most likely transmission source was inferred in only 41 (26%) cases, and the potential for a transmission source outside of the study population was not quantified. Nevertheless, biologically plausible associations between younger age, higher viral load, recent HIV infection, and a recent sexually transmitted infection were found with the probability of being identified as a source of infection.

In the case of incomplete sampling, calculating the probability that a putative transmission pair is real is equivalent to calculating the probability that there are zero unsampled intermediaries between the pair in the viral phylogeny. Calculating this probability is complex, but possible, provided a realistic model of the epidemic process and given good data about incidence and prevalence of infection. This paper is concerned with calculating the probability, henceforth called the *infector probability*, that a given host is the source of infection for another host from phylogenetic and epidemiological surveillance data. The main contribution of this manuscript is the development of a theoretical framework which realistically accounts for the epidemiological and sampling process, thereby correcting for error due to incomplete sampling. This theory also allows for the possibility that the infected population is heterogeneous, such that some individuals have a higher intrinsic infectiousness than others. This is accomplished by the incorporation of patient-level covariates (behavior, stage of infection etc.) into the calculation of infector probabilities.

To demonstrate the utility of infector probabilities to the analysis of real epidemic data, we have simulated a dataset based on the real HIV epidemic among MSM in Detroit, Michigan. Through a simulation-based analysis, we use our solution of the infector probabilities to address the following questions:

- Is it possible to infer transmission events from HIV phylogenetic data with high accuracy?
- Are widely available HIV sequence data collected for drug resistance testing useful for forensic investigations of who infected whom?
- Are estimated infector probabilities useful for epidemiological investigations? Can our methods detect increased transmission rates during early/acute HIV infection (EHI) or other variables that determine heterogeneity in transmission rates?

## Materials and Methods

This section is focused on the derivation of a *n*×*n* matrix of infector probabilities 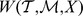 (equation 17) which is a function of

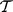
, a binary genealogy with branch lengths in units of calendar time, and

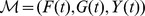
, a process model which provides a timeseries of incidence (*F*), state transitions (*G*) and prevalence (*Y*), and
- *X*, a 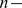
vector that describes the state of each sample unit.

Our solutions employ a population genetic model that assumes that the population size is large, so the model may be biased for very small epidemics or outbreaks. In reality, all of the inputs into our solution of *W* would need to be estimated from real data, which increases uncertainty when identifying transmission pairs. The process model may optionally describe the dynamics of a structured population (a compartmental model), such that each infected individual occupies one of *m* discrete categories. In this situation, *Y*(*t*) is a 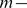
vector valued function of time, and *F*(*t*) and *G*(*t*) are *m*×*m*-matrix valued functions of time. In structured models, 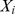 will denote the state (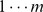) of each sample unit at the time of sampling. Almost all continuous-time compartmental infectious disease models with a discrete state space can be decomposed into the 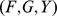 processes [17]. An explicit example of such a decomposition for a realistic HIV model is provided in the Materials and Methods.

Our approach makes use of coalescent theory, which is based on the retrospective modelling of gene tree structure [18]. The state of the tree will be described at a retrospective time *s*, which proceeds from the present to the past. Solving for the state of the tree is accomplished by conditioning on the state of the tree at the tips and working backwards towards the root. An approximation made by the coalesecent model is that the population size 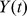 is large, such that the states of lineages in the tree can be assumed to be independent [17]. We assume that hosts *i* and *j* are randomly sampled, or sampling may be stratified according to a categorical variable. We assume that the the phylogeny is reconstructed from a set of sequences that are one-to-one with the set of hosts, i.e. each host is sampled exactly once and has one corresponding pathogen sequence in the phylogeny. Additionally, we will assume that the time of sampling is known for each host, that branch lengths in the phylogeny are proportional to calendar time, and that the tree is bifurcating i.e. that there are no polytomies. For a sample of *n* hosts, our goal is to arrive at an *n*×*n* matrix which gives the probability that a host in the 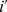
th row transmitted to a host in the 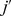
th column. We also employ the same population-genetic assumptions as employed in [17]: 1. Every node in the tree corresponds to a transmission from an infected host to a susceptible host. 2. Each lineage at a single time point corresponds to a single infected host. The first condition is appropriate if viral lineages coalesce very rapidly within hosts relative to their rate of dispersal between hosts. The second condition is appropriate if dual infection is rare (hosts can be infected with at most one lineage at a time). Simulation studies have examined the suitability of these assumptions for HIV [19]. Note that the second condition does not preclude a lineage from passing through multiple unsampled hosts. If sampling is heterochronous (samples occur at multiple time points), we invoke a third assumption: If a lineage is sampled, it does not have descendents which are also sampled. If sampling of direct descendents is allowed, condition (1) would be violated, since a node in the genealogy would correspond to the time of sampling of the ancestral lineage, not a transmission event.

### Infector probabilities in a homogenous population at endemic equilibrium

To give intuition for the method, we first illustrate a simple example of an epidemic within a homogeneous population. The variable *s* will denote time on a reverse axis (time before the last sample is taken), while *t* will represent time on a forward axis (time since some point in the past).

We calculate the probability that host *i* infected host *j* under the conditions that sampling occurs at a single timepoint, and that there is a single type of infected host. We assume that *i* and *j* form a ‘cherry’ (a clade of size 2), and that both the population and sample sizes are sufficiently large such that we can approximate the dynamics of the number of infected individuals in the population, 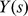, and the number of lineages in the sample, 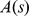, using differential equations. At time 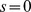, we have 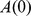 lineages equal to the number of hosts sampled. All of these assumptions will be relaxed in subsequent sections.

What is the probability that *i* transmitted to *j* at their most recent common ancestor, which occurs at time 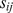
? A necessary condition for this to occur is that the viral lineage at 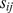 corresponds to virus circulating in host *i*. This condition will not be satisfied if an unsampled individual *k* transmits to *i* before (retrospectively) 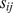, in which case 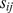 will correspond to a transmission event involving *k*. The rate at which an unsampled host *k* infects *i* at time *s*, 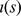 is
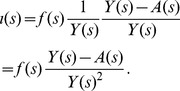
(1)This can be understood as the product of a rate and two probabilities:

- *f* is the rate of new infections in the population.

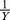
 is the probability that *i* is infected given that a transmission occurs. This is a consequence of the homogeneity assumption, so that every individual has equal chance of being the recipient of a new infection.

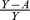
 is the proportion of infected individuals that do not correspond to lineages within the tree. This is also the probability that the host that transmits infection is not represented by a lineage in the tree.

When an unsampled individual *k* transmits to *i*, the viral lineage “jumps” to *k* (recalling that we are considering time from the present to the past). We can therefore count the number of unique infected individuals along the branch that begins at *i* and terminates at 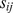. We will denote this random variable 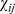, which is given by the following expression:
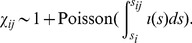
(2)Note that one is added to account for the host *i* itself. We can also calculate the probability of there being no jumps.
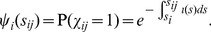
(3)For *i* to transmit to *j*, we must have 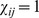 and 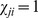. This occurs with probability 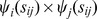. Finally, as infected individuals are homogeneous and sampled at the same time, the probability that *i* transmits to *j* as opposed to *j* transmitting to *i* is 1/2. Hence, the probability that *i* infected *j* at time 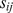 is
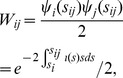
(4)where *W* denotes the matrix of infector probabilities.

To demonstrate how sampling plays a central role in determining the extent to which cherries represent direct transmissions, we will consider a large sample size, such that we can model the number of cherries as well as the number of cherries that correspond to direct transmissions as ordinary differential equations. Previously [20], we have shown that the cumulative number of cherries in a tree at height *s*, 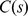 can be written in terms of the rate of coalescence between the leaves of a tree. Let 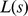 be the number of extant terminal branches of the tree at retrospective time *s* (that is, uncoalesced lineages). We have
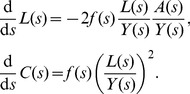
(5)These equations may be understood as the product of a rate (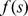) and two probabilities which describe the combination of two lineages at a coalescent event. For example, with probability 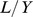, a terminal branch will be involved in a transmission event, and with probability 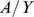 an ancestral lineage will also be involved in the transmission event such that a coalescent will occur. With probability 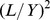, two terminal branches will be involved in a transmission event, and a cherry will form. The total number of cherries in a tree can be calculated by solving for 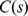 at 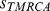, the time of the most recent common ancestor of the sampled sequences.

To determine the number of cherries that represent direct transmission, 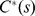, we first derive an equation for the number of leaves of a tree along which no transmissions from an unsampled individual have occurred, 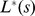, which decreases as a consequence of transmission from others in the sample (as for 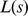) as well as transmission from unsampled individuals:
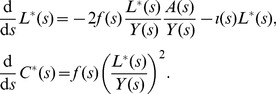
(6)These equations are derived as above, but include an additional hazard 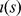 for an unsampled host transmitting to one of the 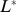 external lineages.

Some analytical insights into how different parameters affect the proportion of cherries that are associated with direct transmission can be obtained under the assumption that the number of infected hosts, *Y*, and the incidence of infection, *f*, are constant, i.e. when the system is at equilibrium, and we drop the time index for these variables. If we define the constant 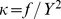, then following [20], the number of lineages over time 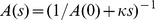, which is a deterministic approximation to the rate of coalescence in a coalescent model of fixed size, and the time to the most recent common ancestor 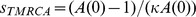, which is obtained by the solution of 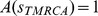. We substitute this expression for 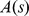 and 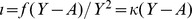 into the equation for 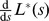 to give the following.
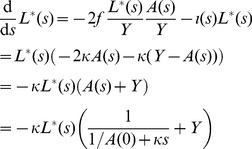
(7)


This can be solved using separation of variables, with the constant of integration calculating by the initial condition 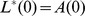.
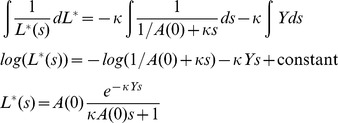
(8)


Substituting this solution of 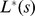 into the differential equation for 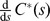 gives the following.
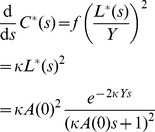
(9)


Solving the above for 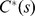 is made more simple using integration by substitution with 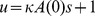 (i.e. changing the timescale), such that 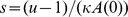, and 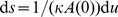.
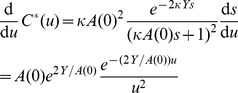
(10)


The solution for 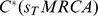, the total number of cherries that represent direct transmission, is found by integrating from 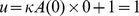 to 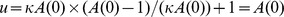 (for 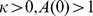). The term 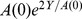 is a constant, and integration of 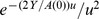 results in an exponential integral term, 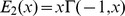, where 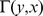 is the upper incomplete gamma function.
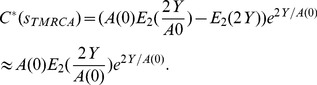
(11)The approximation is for large *Y*, such that 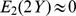.

Similarly, at equilibrium we can substitute 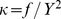 and 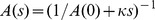 into equation 3, which yields 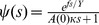. If we know the height of the cherry, 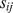, then at equilibrium, the probability that *i* and *j* are a transmission pair is approximately
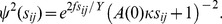
(12)


These results demonstrate that at equilibrium, the fraction of sequences in cherries is independent of sampling fraction (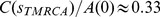), while the proportion of sequences in cherries that represent a direct transmission is a function of the ratio of the number of infected in the population to the number of infected in the sample. Note that even when 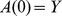, i.e. all individuals have been sampled, not all cherries represent direct transmissions (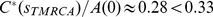). In addition, for more realistic sample fractions, the number of cherries that represent direct transmissions is extremely low; for example, if 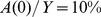, then 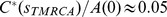.

The very simple expressions in equation 4 and 12 are obtained after applying numerous simplifying assumptions: *i* and *j* are sampled at the same time, *i* and *j* are monophyletic, and the epidemic is at equilibrium (*Y* and *f* are constant). In the next section, we proceed to relax all of these assumptions. Nevertheless, equation 4 may be a good approximation in some situations when 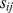 is close to the time of sampling and if incidence and prevalence is relatively constant between 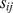 and the time of sampling.

### General framework for derivation of infector probabilities

The solutions described below are applicable to a large class of infectious disease process models which describe the incidence and prevalence of infection over time. The host population is not assumed to be homogeneous, but can have arbitrary discrete structure. Each infected host can occupy any of *m* states (a compartmental model), and an infected host cannot transmit to more than one susceptible at a single point in time.

The discrete states that a host may occupy will be indexed by variables *k* and *l*. Under these conditions, the model can be decomposed into the following processes (see [17] for details):

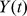
: 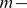
vector valued function of time which describes the number of infected hosts at time *t*.

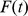
: 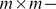
matrix valued function of time which describes the rate of transmission from each state to each other state. 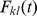 describes the rate that all infected hosts in state *k* generate new infections in state *l*. This does not imply that the transmitting individuals move to state *l*; transmission may occur across categories, for example, men infecting women. This will also be called the *birth* matrix.

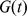
: 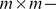
matrix valued function of time which describes the rate of transition from each state to each other state. Several examples of processes that would be included in *G* are: progression through discrete stages of infection, diagnosis, discrete changes in risk behavior, and geographical migration. This will be called the *migration* matrix.

The process model will be denoted by the tuple 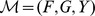. An explicit example of decomposition of a model into 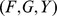 is given for an HIV model below.

In [17], master-equations were developed for computing many attributes of 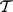 conditional on 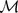, such as the likelihood. This approach can, for example, be used to fit models to phylogenetic data. A similar approach is taken here, and we will re-use notation where possible. The primary aim is to derive 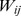, the probability that a host *i* directly transmitted infection to host *j* based on phylogenetic data (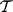). The master equations will describe the dynamics on a reverse time axis. In common with other coalescent models, our solutions will work by integration from the present to the past along the reverse time axis *s*. The variables 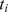 and 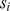 will denote the times of sampling of host *i*, and the initial conditions will be based on the states of hosts at these times.

**Figure 2:**
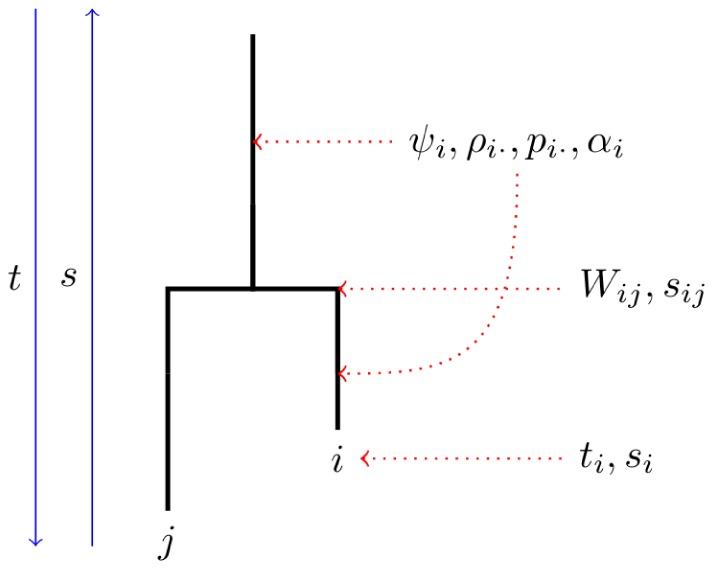
A schematic of a gene tree with variables of the coalescent model corresponding to tips, branches, and nodes of the tree.

The coalescent model described here is complex, so a visual aid is provided in Figure 2. A useful way to conceptualize the organization of this model is to visualize every branch in 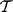 as having a set of dynamic variables associated with it for every tip of the tree descended from it. Every node will have associated with it the probabilities 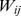 for every pair of sampled hosts 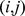 descended from it.

At some point in the past, every sampled host *i* has an ancestral host; in other words, the ancestral host harbors virus which is ancestral to the virus that is sampled from host *i* at 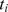. We will denote the ancestral host of *i* as 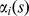. Note that we may have 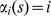 if *i* became infected at a retrospective time 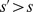, in which case the ancestral branch of *i* in 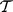 at time *s* corresponds to the host *i* itself. The variable 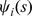 will denote the probability of this event. 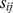 will denote the time of most recent common ancestry for virus sampled from hosts *i* and *j*. 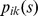 will denote the probability that 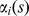 is in state *k*. The master equations describing evolution of 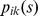 were derived in [17]. Here, we introduce a similar variable 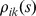, which is the probability that 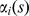 is in state *k* conditional on 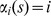.

Derivations of 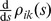 and 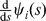 are provided in subsequent sections. Here, we show how 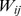 is calculated when 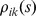 and 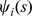 are known.

Consider the node in 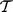 corresponding to the MRCA of *i* and *j* at time 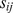. In order for *i* to transmit to *j*, we must have 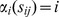 and 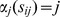, i.e. both daughter lineages of the node correspond to hosts *i* and *j*. The probability of this event is 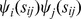, since events are assumed to be independent. That is a good approximation when *Y* is large. At 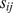, the states of host *i* and *j* are described by the vectors 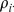 and 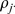.

Suppose that at 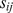 a type *k* host transmits to a type *l* host, which occurs at rate 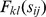. The probability that host *i* is the transmitter conditional on 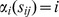 is 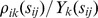, i.e. the probability that *i* is selected from the 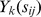 infections of type *k* and the probability that *i* is type *k*. Similarly, the probability that *j* is the recipient of infection conditional on 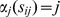 is 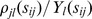, i.e. the probability that *j* is selected from 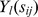 infections of type *l* and the probability that *j* is type *l*. Considering all possible types of transmission *k* and *l*, the rate that *i* transmits to *j* is as follows.
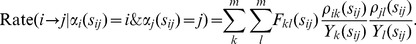
(13)This can be written with greater economy using matrix notation.
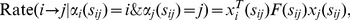
(14)where 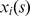 is an 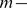
vector with elements 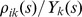. Similarly, the rate that *j* transmits to *i* is as follows.
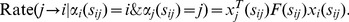
(15)If both daughter lines correspond to *i* and *j*, a transmission must have taken place between them. The probability 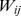 is obtained by taking the ratio of the rate that *i* transmits to *j* to the rate that transmission occurs in either direction.
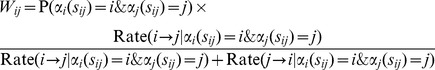
(16)

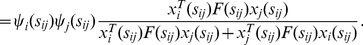
(17)


### Derivations of master equations for *ψ* and *ρ*

The function 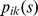 describes the probability that the ancestral host of the sampled host *i* is in state *k* at retrospective time *s*. Equations for the dynamics of 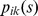 are derived in [17]. Here, we derive similar equations for 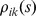, which describes the probability that the ancestral host of *i* is type *k* at time *s* conditional on *i* being the ancestral host; in other words, the branch in 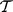 that is ancestral to *i* at time *s* corresponds to the host *i* itself (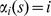). It is assumed that at each time of sampling 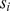 we know the state of *i*; this information provides the initial conditions for the set of equations that describes the dynamics of 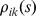.

Suppose that at retrospective time 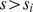, 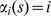 and *i* is in state *k*. In a small time step *h*, approximately 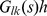 infected hosts will migrate from state *l* to state *k*. Then retrospectively, the probability that host *i* will change state from *k* to *l* is approximately 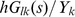, where the factor of 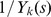 is the probability of selecting *i* if drawing a single individual from 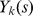 infected hosts. Considering the limit 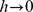, this leads to the following equations.
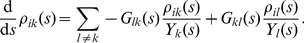
(18)


In matrix notation, the derivative of the vector 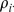 can be expressed as
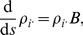
(19)where *B* is a *m*×*m* matrix with elements:
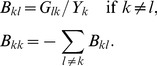
(20)


Suppose that at a time 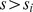, there is a node in 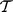 at the branch that is ancestral to *i*. At a node, 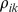 undergoes a discrete change as we incorporate information about the state of the other daughter branch at the node. Let 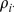 and 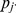 represent the two state vectors for two daughter branches of the node at the MRCA of *i* and *j*, which occurs at retrospective time 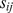. Note that we will use the state vector 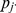 for the ancestral host of *j*, since we are not conditioning on the event that *j* corresponds to a daughter branch at 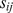. Under the assumptions of this model, a transmission event occurs at this node, either from 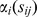 to 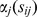 or vice versa. The discrete change at 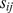 will occur after an infinitesimal time 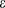. In order for the event 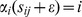 to occur, *i* must be the transmitter at the node. Hence, the probability 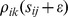 is simply the probability that the transmission is made by a type *k* conditional on *i* being the transmitter. This is
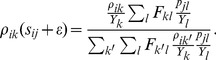
(21)

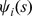
 is the probability that 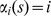. Equations governing 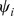 are found by considering the hazard of an ‘invisible transmission event’ [20], [21], which changes the ancestral host of a branch in the phylogeny without producing a coalescent event. Equations for *ψ_i_* will have a continuous component for branches and a discrete component for nodes.

Suppose that at retrospective time *s*, 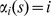 and *i* is in state *k*. 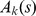 will denote the number of ancestors of the sample at retrospective time *s* that are in state *k*. Following the approach taken in [17], the rate that a transmission 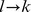 leads to a change of 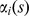 is
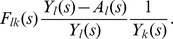
This is the product of the rate of transmissions 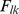, the probability 1/*Y_k_* that *i* is selected as the recipient of transmission, and the expression 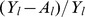, which is the probability that the transmitter is not ancestral to the sample (i.e. that no branch in the tree corresponds to the transmitting host).

This motivates the following equation for the derivative of *ψ_i_*:
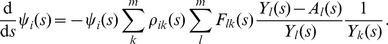
(22)


At an ancestral node of *i*, *ψ_i_* undergoes a discrete change by a factor which is simply the probability of *i* being the host that transmitted at the node:
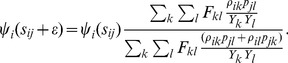
(23)


Software for calculating *W_ij_* as described in this paper is available at http://code.google.com/p/colgem/.

### Simulations

We simulated HIV gene genealogies using an individual-based stochastic simulation based on the epidemic model presented in [19]. These simulations were carried out with the objective of replicating a real HIV dataset as closely as possible, while allowing us to know who infected whom. Sample sizes, the times of sampling, and incidence of infection were all chosen to coincide as closely as possible to the dataset of HIV sequences described in [19], which was based on 662 HIV-1 sequences sampled from men MSM in the Detroit metropolitan area. Simulated sequences and estimated phylogenies were also chosen to mimic the diversity expected for a sample of subtype B sequences. To capture heterogeneity in simulated outcomes, 20 independent simulations were undertaken.

**Figure 3:**
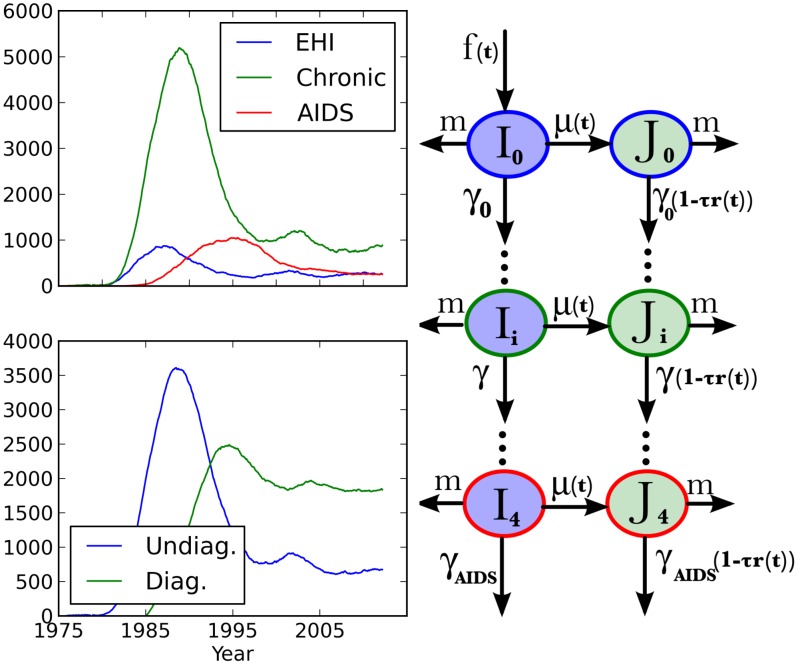
Model used to simulate HIV phylogenies. Left: Simulated number of infections over time. Infections are aggregated by stage of infection (top) and by diagnosis status (bottom). Right: Flow-diagram showing the progression of infected individuals through 5 stages of infection, diagnosis, and death. The color of compartments correspond to diagnosis status in prevalence figures on left. The color of outlines corresponds to stage of infection in prevalence figures on left. The per-capita rate of state transitions is shown over arrows.

The HIV model is illustrated in Figure 3. The model in [19] was fitted to a combination of surveillance timeseries data, such as HIV/AIDS diagnoses over time and HIV genetic sequences. This provided an estimate of incidence and prevalence over time as well as estimate of the number of transmissions made by infected individuals in different stages of infection. Parameter estimates in the simulations were taken from the maximum likelihood model fit in [19]. In this model, infected individuals progress through five stages of infection and can be undiagnosed or diagnosed. Diagnosed individuals may additionally receive antiretroviral therapy (ART) which reduces the rate of progression towards AIDS and death. We assume that ART is available after 1998 to all diagnosed individuals. Chronic infections transmit at rate 87.5% smaller than the transmission rate of early HIV infection (EHI), and there are no transmissions from AIDS cases oweing to effective diagnosis and treatment. In this model, the first stage of infection, EHI, lasts 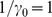 year, three chronic stages last 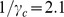 years on average each, and AIDS lasts 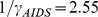 years on average. The total infectious period may be much longer with treatment, which is largely determined by natural mortality, which occurs at the rate *m*(*t*) of 1 per 27 years.

An essential aspect of this model is how incidence *f*(*t*) and diagnosis rates *μ*(*t*) vary over time. In this model, both of these rates are described by spline functions, and we re-use the parameters of the spline functions estimated in [19].

In the discrete individual-based simulations, the time to the next transmission event is exponentially distributed with rate *f*(*t*). We make the approximation that *f*(*t*) is constant between transmission events, which is a good approximation since the time between transmissions in the population is quite short relative to the change in *f*(*t*). At each transmission event, the transmitting individual is selected randomly from the set of all infected individuals with a weight that depends on the stage of infection of the individual and whether they are diagnosed. For example, someone with undiagnosed chronic infection will transmit at a rate less than an undiagnosed EHI by a factor of 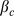 as described above, and a diagnosed chronic infection (pre-treatment) will transmit at a rate less than an undiagnosed EHI by a factor of 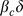. Similarly, the time to the next diagnosis event is exponentially distributed with rate *μ*(*t*), and the newly diagnosed individual is selected uniformly at random from the set of all undiagnosed infections.

Note that the the simulation may be put in the canonical form 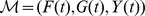 described above, which allows simulations to be used to calculate infector probabilities. In this case, *m* = 10 (infected may occupy 5 stages and be diagnosed/undiagnosed), and *Y*(*t*) is an 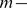
vector that describes how many infected are in each state at time *t*. 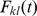 gives the transmission rate from state *k* to *l* at time *t*, so for example, if *k* corresponds to undiagnosed chronic infection, and *l* corresponds to undiagnosed EHI, 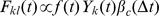, where 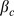 is the relative infectiousness of chronic infections. 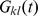 represents the rate that type *k* changes state to type *l*; in this model, this process corresponds to stage progression and diagnosis. For example, if *k* corresponds to undiagnosed EHI and *l* corresponds to the first chronic stage, then 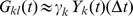.

To reconstruct a gene genealogy from the simulation, we iteratively build a binary tree by adding a new branch at each transmission. The logic underlying tree reconstruction is given in [21]–[23]. Briefly, if an individual *z* transmits at time *t*, we add a new branch to the tree which connects a new node *u* with an old node *v*. Each node in the tree has a time associated with it. The time of *u* is the time of the new transmission event *t*. The node *v* that is connected to *u* corresponds to the last transmission event that involved host *z*. That event may be another event in which *z* transmitted, or it may correspond to the event where *z* became infected. All of the internal branch lengths in the tree therefore correspond to the time between consecutive transmission events.

In reality, we do not observe the complete transmission genealogy, but rather a small subsample. To model sampling, we randomly sampled *n* = 662 branches heterochronously at regular intervals between the 29th and 37th year of the epidemic. At each sampling time, we introduce a terminal node into the tree with a corresponding time of sampling. The sample size and sample window were chosen to mimic the real dataset in [19]. Unsampled branches are then pruned from the tree, which yields a final binary tree with *n* terminals and 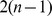 internal branches.

As noted above, the calculation of 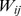 in heterochronous samples does not account for the possibility that a sampled lineage is a direct descendent of a previously sampled ancestral lineage. Nevertheless, we allow this event to occur in simulations in order to evaluate if violation of this assumption is a large source of bias.

### Sequence simulation and phylogenetic analysis

To simulate genetic sequence alignments corresponding to the simulated genealogical relationships described in the previous section, we used the program Seq-Gen v.1.3.3 [24]. For each simulated tree, we generated a sequence alignment of 662 sequences, each 1200 nucleotides in length. We used an HKY nucleotide substitution model with a transition-transversion ratio of 4.73, and rate heterogeneity modeled as a mixture of invariant sites (47%), a mean substitution rate of 1.6e-03 per site per year, and a Γ distribution discretized into four categories with a shape parameter of 0.714. These parameters were obtained by a previous phylogenetic analysis of real HIV data [25].

For each sequence alignment, we used relaxed-clock Bayesian methods [26] as implemented in the software BEAST [27] to estimate a posterior distribution of phylogenetic trees. We assumed a GTR substitution model, with rate variation modeled as a mixture of invariant sites and four-category discretized Γ distribution. We used the semi-parametric skyride method [28] to estimate how the effective population size changes through time. Parameters were estimated using a Markov Chain Monte Carlo algorithm which was run for 50 million iterations. We discarded the first 50% of samples as burn-in. To generate estimated infector probabilities from the posterior distribution, we calculated 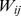 for a sample of 50 trees and report the mean. We also compare 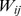 between samples from the BEAST posterior to investigate uncertainty in 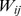 oweing to uncertainty in the underlying phylogeny.

Results for the HIV model presented below which are based on a true transmission genealogy utilize 20 independent simulations. Results that utilize simulated sequences are based on only a single simulation, but utilize 50 posterior sampled trees.

### Simulations for model validation

Text S1 and figures S1, S2, S3 describes several additional simulation experiments to validate the numerical accuracy of the approach. These simulations were undertaken using idealized compartmental SIRS models with homochronous samples. The time of sampling (peak prevalence of endemic equilibrium) was investigated.

Define
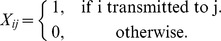
In the absence of bias, the expected residual 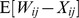 should be zero where the expectation is taken across all pairs 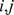 in all simulations. A t-test was performed to test the hypothesis that 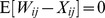.

Code for all simulation experiments can be found at https://code.google.com/p/inferring-the-source-of-transmission-with-phylogenetic-data/.

## Results

Synthetic HIV datasets were generated which matches the data described previously in [25] and [19]. This dataset comprised an alignment of 662 HIV-1 subtype B partial-*pol* sequences originally collected for drug resistance testing. All sequences were collected within one year of diagnosis from treatment-naive individuals with self-described MSM risk behavior. Sequences were sampled heterochronously over the period 2004–2012. Additionally, associated with each sequence are clinical covariates such as CD4 counts, last negative test dates, and BED test results [19] that are informative about the stage of infection at the time of diagnosis. In the simulation results, we assume that the stage of infection is known for each sample unit.

The number of HIV infections over time are shown in Figure 3 for a single simulation. These trajectories are similar to maximum likelihood estimates obtained in [19] for MSM in the Detroit Metropolitan area. At the end of 2011, there are 2509 prevalent infections according to this simulation, and approximately 662/2509 = 26% of these are sampled for phylogenetic analysis.

**Figure 4:**
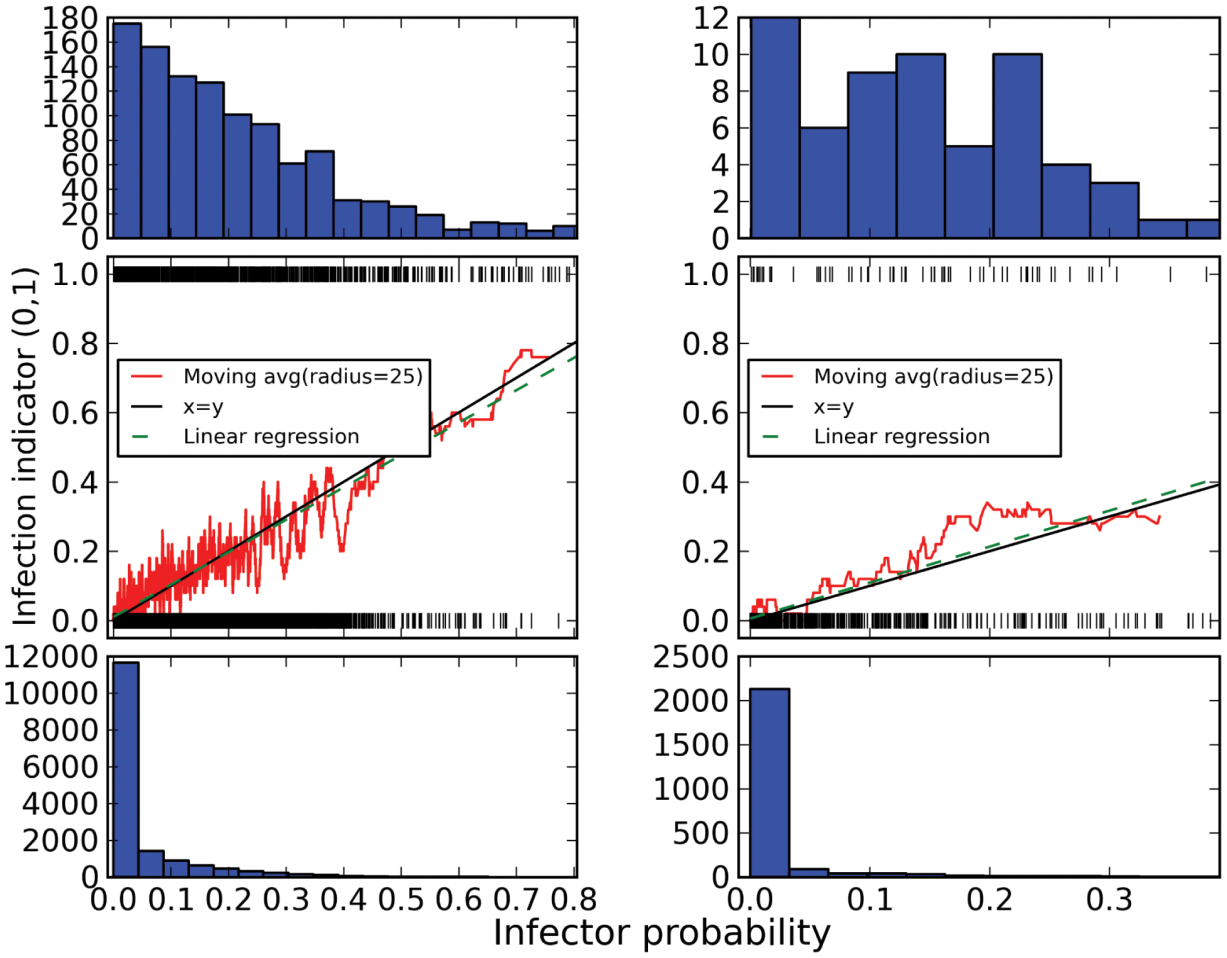
Comparison of infector probabilities and frequency of transmission events in simulations. On the left, infector probabilities are calculated for the true transmission genealogy in 20 independent simulated HIV epidemics and samples of 662 individuals. On the right, infector probabilities are based on simulated sequence data for a single simulation and a sample of 662 individuals. Data are pooled from 50 trees sampled from the Bayesian phylogenetic posterior distribution. Middle: The estimated infector probabilities (x-axis) versus whether a transmission actually occured (hash marks) for all pairs of sampled individuals in the HIV simulation. The red line shows a local-average of the frequency of transmission events. The green line shows a linear regression of true transmission events (coded zero or one) on the estimated infector probability. Histograms show the frequency of estimated infector probabilities when transmissions happen (top) and when they don't (bottom).

Infector probabilities for the HIV simulations are shown in Figure 4. We compare estimates based on the true transmission genealogy, which is not generally known in applications with real data, and a sample of phylogenies from the Bayesian phylogenetic posterior distribution estimated from simulated sequences. Estimates for the true genealogies are based on pooled results for 20 independent simulations, while estimates for the estimated phylogenies are based on a single simulated sequence alignment and 50 trees sampled from the BEAST posterior distribution. Infector probabilities were calculated and compared for all possible pairs of sampled individuals. With both estimated and true genealogies, we find that the infector probabilities increase at the same rate (slope≈1) as the frequency of true transmission events, which are known from the simulations. We regressed the known transmission events, coded as zero or one, on the estimated infector probabilities. If the infector probabilities perfectly coincide with the true frequency of transmission events, the slope of the regression line will be one and the intercept zero. The slope and intercept for the regression line calculated from the true genealogy are respectively 0.93 and 0.01. The slope and intercept of the regression line calculated from 15 estimated phylogenies are respectively 1.04 and 0.006.

Histograms in Figure 4 also show the frequency of transmission events stratified by the estimated infector probability. This shows that the infector probability is generally quite close to zero in the majority of cases that a transmission event actually occurred. In almost all cases where a transmission did not occur, the estimated infector probability is very close to zero. Considering all 20 simulations, there are 
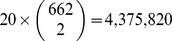
 possible transmission events given a sample of 662, and we observed only 1,079 transmission events. Thus, there is only 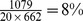 probability that the donor of a random patient also appears in the sample. This probability depends on details of the epidemiological model, which types of individuals are sampled, and when samples are collected. In this instance, the probability is much lower than the sample fraction (approximately 26%) since the sample is collected over time and the donor for many cases are diagnosed or deceased (only undiagnosed cases are sampled).

**Figure 5:**
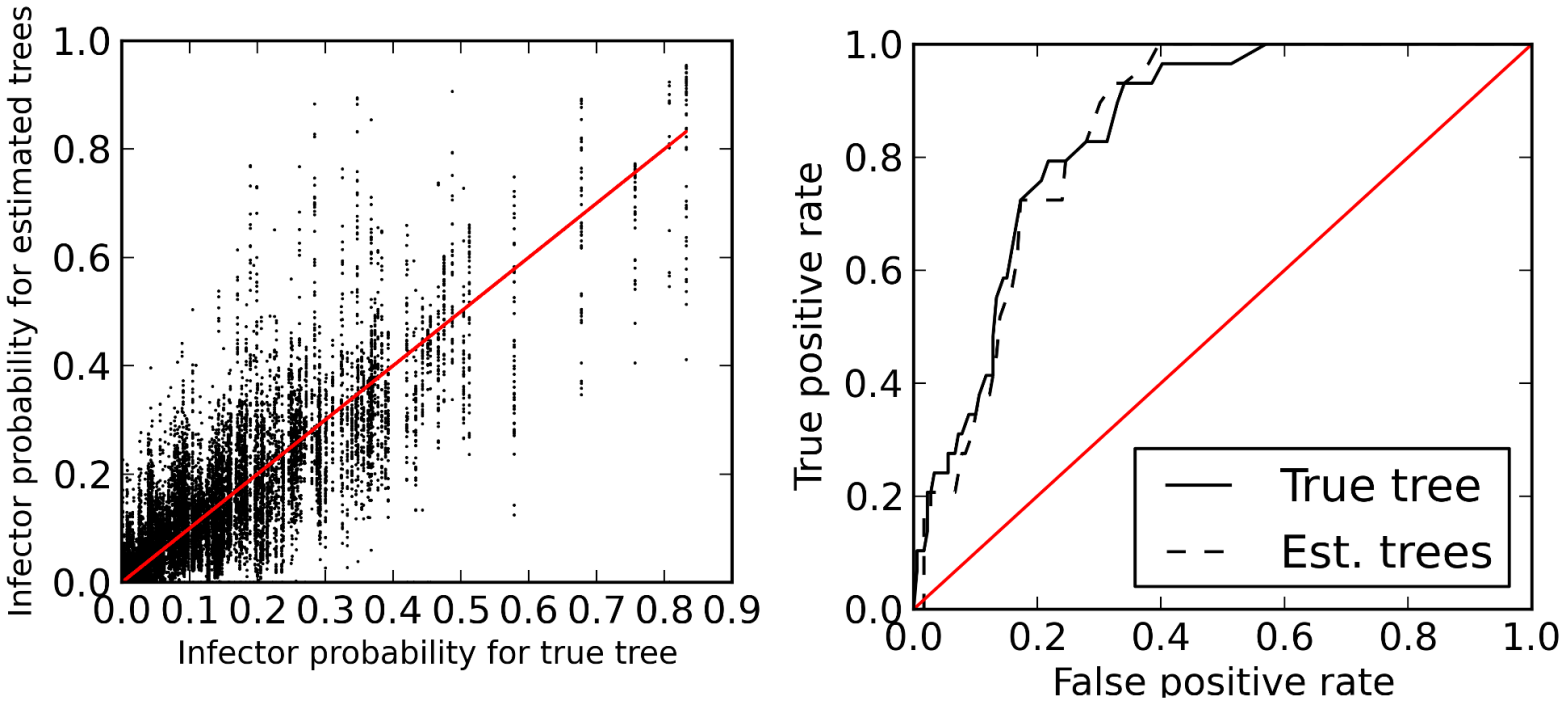
Performance of estimated infector probabilities. Left: Estimated infector probabilities based on the true transmission genealogy versus infector probabilities based on a sample of trees from the Bayesian phylogenetic posterior distribution. The red line shows 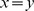. Right: True positive versus false positive rates (ROC) using estimated infector probabilities for classification of who infected whom in simulated HIV epidemics. The ROC curves were calculated for 208 pairs of individuals clustered in cherries in the transmission genealogy. Estimates are shown for the true transmission genealogy for a sample of 662 individuals and for the average infector probability calculated from a sample of 50 trees from a Bayesian phylogenetic posterior distribution.

Figure 5 shows estimated infector probabilities based on the true transmission genealogy from a single simulation and on 50 trees sampled from the Bayesian-phylogenetic posterior distribution for a single simulated multiple sequence alignment. The Pearson correlation coefficient between these two sets of infector probabilities is 83%.

Figure 5 also shows true positive and false positive rates (ROC) if estimated infector probabilities are used for classification of the event that a prospective transmission pair is real. The data are based on a single simulation of the HIV model and a single simulated sequence alignment. If we consider the set of all potential transmission pairs, the classification of true negatives will generally be extremely accurate because distant pairs in the tree will have very low infector probabilities; consequently, the false positive rate will be extremely low for all but the smallest threshold values. Therefore, we confine the analysis to a more difficult problem of identifying true transmission pairs in the set of 208 potential transmission pairs corresponding to cherries (2-clades) in the true genealogy. ROC curves are shown for infector probabilities based on the true transmission genealogy and on the infector probability averaged across 15 trees sampled from the Bayesian-phylogenetic posterior. Both ROC curves have similar properties; the area under the curve (AUC) is 84.7% for the estimated phylogenies and 84.8% for the true genealogy.

Comparing aggregated infector probabilities can be used to detect systematic differences in transmission rates between categories of infected individuals. Relative values of infector probabilities are not equivalent to relative transmission rates, and these statistics should not be interpreted as estimates of relative transmission rates. But, we do expect that relative infector probabilities to trend in the same direction as transmission rates. Figure S4 compares infector probabilities for different stages of infection and for undiagnosed versus diagnosed individuals. These results are based on a single simulation of the HIV model and a single sequence alignment and use a sample of 15 trees from the BEAST posterior distribution. For individual *i*, the expected number of transmissions to other individuals in the sample is the sum of the infector probabilities: 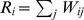. Individuals who have been infected longer are expected to have larger 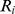, however by dividing by the time since infection 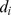, we may detect increased transmission during EHI. An adjusted infector probability for each category is found by dividing each 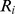 by the expected duration that *i* has been infected given that they are sampled in each category:
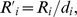
We use the approximation that 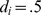 years if *i* is sampled with EHI, 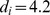 years if *i* is sampled with chronic infection, and 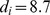 years if *i* is sampled with AIDS. Figure S4 shows stark differences in the number of transmissions attributable to different categories of infected individual.

In the simulations, EHI transmit at a greater rate than chronic infections by a factor of 12.4. If we compare medians of 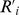, we find transmissions from EHI relative to chronic by a factor of 17.2. In the simulations, undiagnosed individuals transmit at a greater rate than diagnosed individuals by a factor of 12.1 after 1998 and by a factor of 6.4 before 1998 because of the effects of treatment. Comparing medians of 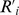, we find transmissions from undiagnosed relative to diagnosed by a factor of 7.2.

**Figure 6:**
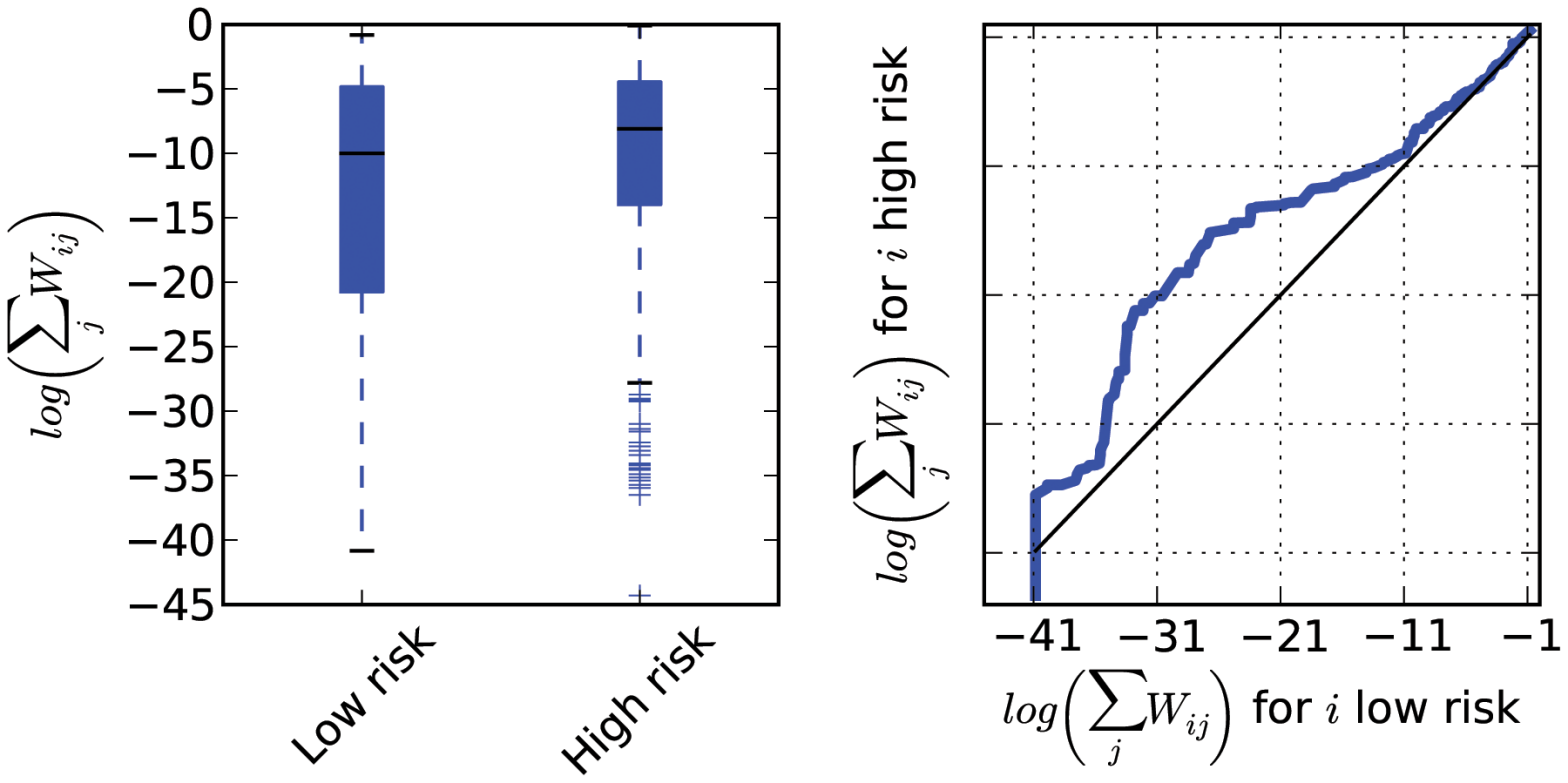
Left: The log of the expected number of transmissions to at least one other sample unit is shown in aggregated form for two risk groups. The high risk category transmits at a rate 10× that of the low risk category. Right: A quantile-quantile comparison of the distributions of log infector probabilities. A quantile-quantile comparison for undiagnosed and diagnosed is shown at bottom right.

To demonstrate the feasibility of detecting covariates that impact transmission rates that are not explicitly included in the calculation of *W*, we conducted another simulation that was identical in all respects to those described above except that half of infected individuals (the ‘high risk’ group) transmit at a rate that is 10× greater than the other half (the ‘low risk’ group). Susceptibility was not correlated with infectiousness in this simulation. We then calculated *W* and 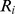, and these values are compared for high and low risk groups in Figure 6. Infector probabilities are much greater for those in the high risk group. The median of 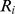 in the high risk group is greater than in the low risk group by a factor of 6.7.

### Model validation

To validate the numerical accuracy of our derivation of 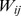, we present additional simulations in Text S1. In these emperiments, more simulations are carried out and more transmissions are observed so that estimated infector probabilities can be compared with a large sample of transmission events.

In all, 1158 SIRS epidemics were simulated, 59194 potential transmission pairs were evaluated, yeilding 3168 within-sample transmission pairs. A sample of 5% of infections was taken at peak prevalence and endemic equilibrium. Bias was not detected for either sampling time (t-test 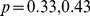).

## Discussion

We have presented a method for calculating the probability that one host infected another (the infector probability) in a pathogen phylogeny. This method makes use of extra epidemiological information, such as the incidence and prevalence of infection over time. The method thereby accounts for the possibility that unsampled infected individuals act as either intermediaries or as a common source of infection for a putative donor and recipient of infection. Any infectious disease model that is used to estimate incidence and prevalence of infection implies a relationship between pathogen gene genealogies and infector probabilities. This is the first method which makes the connection between infector probabilities, infectious disease models, and pathogen genealogies explicit. The practical importance of this method is that it enables the estimation of infector probabilities in situations where there is incomplete sampling, which is more often than not the case for high-prevalence community-acquired pathogens like HIV.

Once 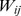 is calculated, a variety of auxiliary analyses are enabled. The column sum 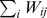 is equivalent to the probability that the infector of *j* is in the sample. This statistic will be sensitive to the number of patients sampled and the times of sampling. The row sum 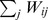 is equivalent to the expected number of secondary infections for case *i* which also appear in the sample. Variation of this statistic can be examined with respect to covariates that may influence transmission rates. Such investigations may indicate which clinical, demographic, and behavioral variables have a large impact on transmission rates and thereby guide further model development.

We have also demonstrated the method using a simulated HIV dataset in which we know who actually infected whom. The dataset was designed to mimic a real HIV dataset, both in terms how patients are sampled and in the epidemiology of infection in the simulated community; phylogenies were estimated from simulated sequences in order to realistically reproduce phylogenetic error. The method is subject to bias due to finite population size and violation of model assumptions. Nevertheless, we have not detected substantial bias in realistic simulation experiments, which suggests that bias will be quite small for applications provided an appropriate epidemiological model is used. Figure 4 shows that accuracy is not greatly impacted by phylogenetic uncertainty stemming from the simulated sequences in this application. Although there is very high variation in estimated infector probabilities between individual trees in the Bayesian-phylogenetic posterior distribution, the infector probability averaged over a sample of phylogenies has similar performance to infector probabilities calculated from the true tree. As Figure 5 shows, infector probabilities calculated from the true tree are highly correlated with estimated phylogenies, but on an individual basis, there can be huge discrepancies. For example, according to the true tree, an infector probability may be 90%, while according to an estimated tree it may be as low as 35%. Due to the potential for false positive classification, which may occur even if the true genealogy is known, it is more concerning that probabilities calculated from estimated trees can also be much greater than those based on the true tree.

It is also important to note that this simulation study assumed perfect knowledge of incidence and prevalence of infection over time as well as perfect knowledge of the stage of infection at the time each infected host is sampled. In reality, there will be substantial uncertainty regarding both, and that would add additional error to estimated infector probabilities. Even though there is very high variance in the infector probabilities based on estimated phylogenies, the infector probability averaged across estimated phylogenies has similar performance as a statistic for classification (AUC of ROC).

There has been controversy [29]–[31] regarding whether abundant HIV sequences collected for clinical purposes may be useful for forensic investigations into who acquired infection from whom. Alternatively, such sequence data may be useful for epidemiological investigations only. An obvious temptation is to use the proposed models in forensic cases. At realistic levels of sampling that resemble currently availabe HIV DRM sequence databases, infector probabilities are quite small. In other words, even though the method may give a realistic estimate of the probability that *i* infected *j*, we rarely have much confidence that *i* infected *j*. In addition, forensic investigations often employ a more targeted approach to sampling and serial sampling of individual hosts [7], [29], which violates the assumption of simple random sampling used in our models. Calculating infector probabilities may actually be helpful for protecting patient confidentiality, since sequence data could be screened and stripped of closely linked pairs prior to being deposited in public databases.

Our simulation experiments have demonstrated how infector probabilities are sensitive to many factors in addition to the structure of the phylogeny, such as details about who is sampled, when they are sampled, and the state of infected individuals at the time of sampling. Details of the epidemic process such as incidence and prevalence over time also influence infector probabilities. Most clustering methods employ a threshold genetic or evolutionary distance, but, as shown in Figure S5, there is a noisy relationship between infector probabilities and the cophenetic distance within the HIV gene genealogy. Infector probabilities are highly correlated with phylogenetic distance, yet for a given phylogenetic distance, the infector probabilities may differ by many orders of magnitude. Getting a realistic picture of potential transmission pairs requires consideration of all of the factors included in our solution for the infector probabilities.

Even though transmission events could not be inferred with high confidence, the application of infector probabilities to epidemiological investigations of HIV seems promising in light of the results in Figure 6 and S4. Infector probabilities capture increased transmissions by those with early infection and those who are undiagnosed at the time of sampling relative to those who are diagnosed. We can also detect the effects on transmission rates of covariates that are not explicitly included in the coalescent model.

Our models have additional utility beyond the calculation of infector probabilies. Similar methods could be used to calculate the distribution of the number of unsampled infected individuals in a transmission chain between two sample units. For example, this has relevance for studies of the evolution of virulence of HIV [32], [33], which is frequently assessed by conducting comparative phylogenetic analyses of set-point viral load and declining slope CD4. Most comparative phylogenetic analyses are based on diffusion models of a continuous trait, however models which account for discrete transmission events may be more appropriate. One could, for example, use information about the length of a transmission chain to obtain estimates of how set point viral load correlates between epidemiologically linked pairs.

This method for calculating infector probabilities is based on a population genetic model that makes assumptions about the epidemiological and immunological process. The model does not account for the potential for superinfection, recombination, or complex within-host evolutionary dynamics which could confuse phylogenetic inference and decrease confidence in putative transmission links. Furthermore, the model does not account for multiple- or serial-sampling of a single infected host. Future research is needed on methods for relaxing these assumptions as well as for quantifying error that may arise from violation of model assumptions in realistic settings.

## Supporting Information

Figure S1 Simulated number of infections through time for the SIRS model.
(PNG)

Figure S2Regression of true transmission events (ticks on axis coded zero or one) on calculated infector probabilities 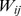. Left: samples of 10% at endemic equilibrium. Right: samples of 10% at peak prevalence.(PNG)

Figure S3 Top: Model structure and population size over time for a model with three states. Blue arrows represent birth within and between states. Red arrows represent migration between states. Bottom: Regression of true transmission events (ticks on axis coded zero or one) on calculated infector probabilities. At right is shown the ROC curve if infector probabilities are used for classification of the event that a putative transmission pair is real.
(PNG)

Figure S4 The log of the expected number of transmissions to at least one other sample unit is shown in aggregated form for different stages of infection and diagnosis status (top). Each stage is represented twice in this figure because an infected individual may be undiagnosed or diagnosed (labels prefixed with ‘D.’). A sampled lineage from an undiagnosed individual corresponds to a situation in which a pathogen is sequenced at the same time that the patient is diagnosed. A quantile-quantile comparison of the distributions of log infector probabilities for EHI and chronic stages is shown at bottom left. A quantile-quantile comparison for undiagnosed and diagnosed is shown at bottom right.
(PDF)

Figure S5 The cophenetic distance between each pair of tips in the HIV gene genealogy is shown versus the calculated infector probabilities.
(PNG)

Text S1 Additional simulation experiments.
(PDF)
