# Supplementary figures and images for: Inferring the Source of Transmission with Phylogenetic Data
Source: PLoS Comput Biol. 2013 Dec 19;9(12):e1003397. doi: 10.1371/journal.pcbi.1003397 (PMC3868546; doi:10.1371/journal.pcbi.1003397)

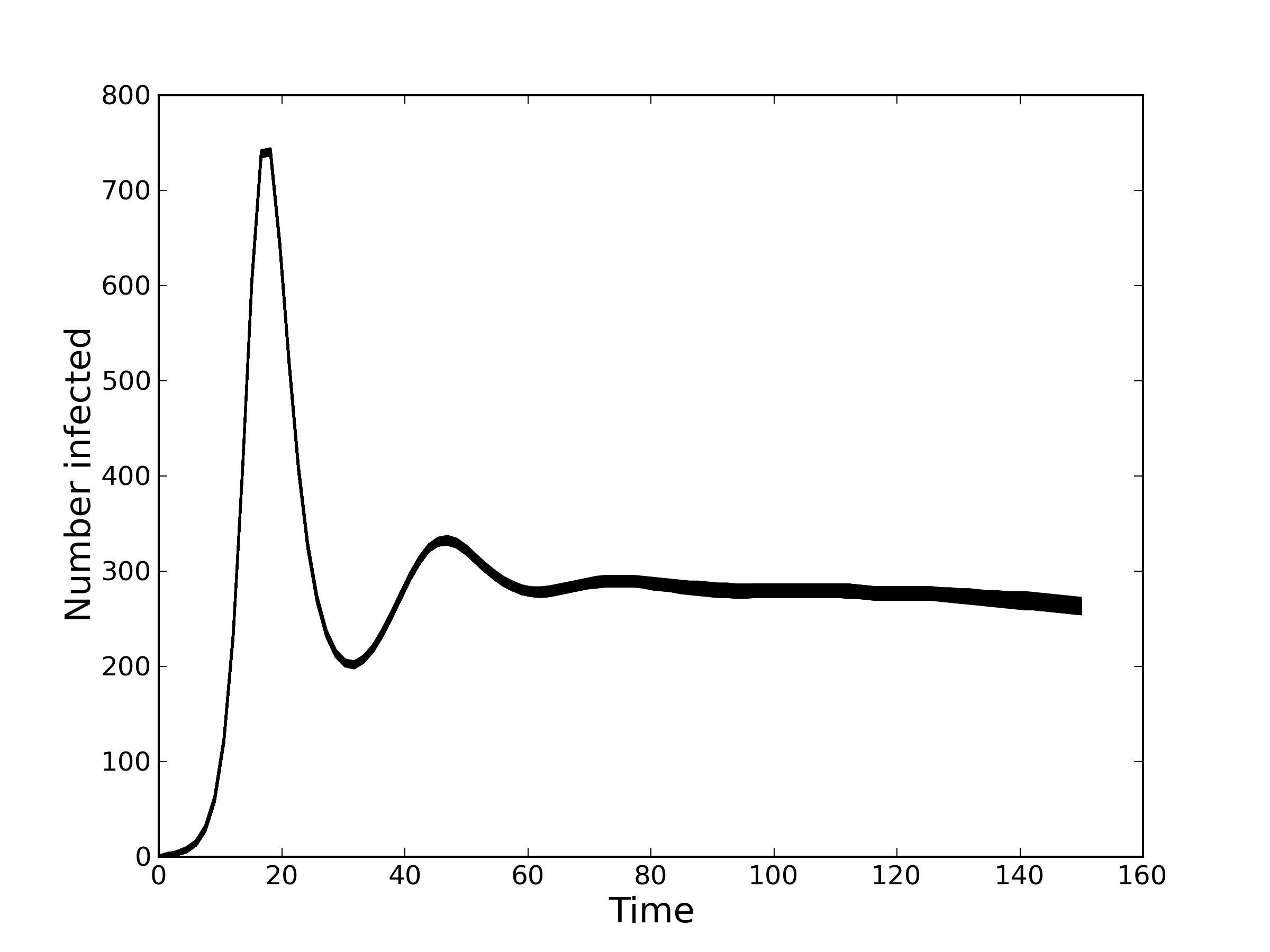

Supplement: Figure S1 — Simulated number of infections through time for the SIRS model. (PNG) [file pcbi.1003397.s001.png]

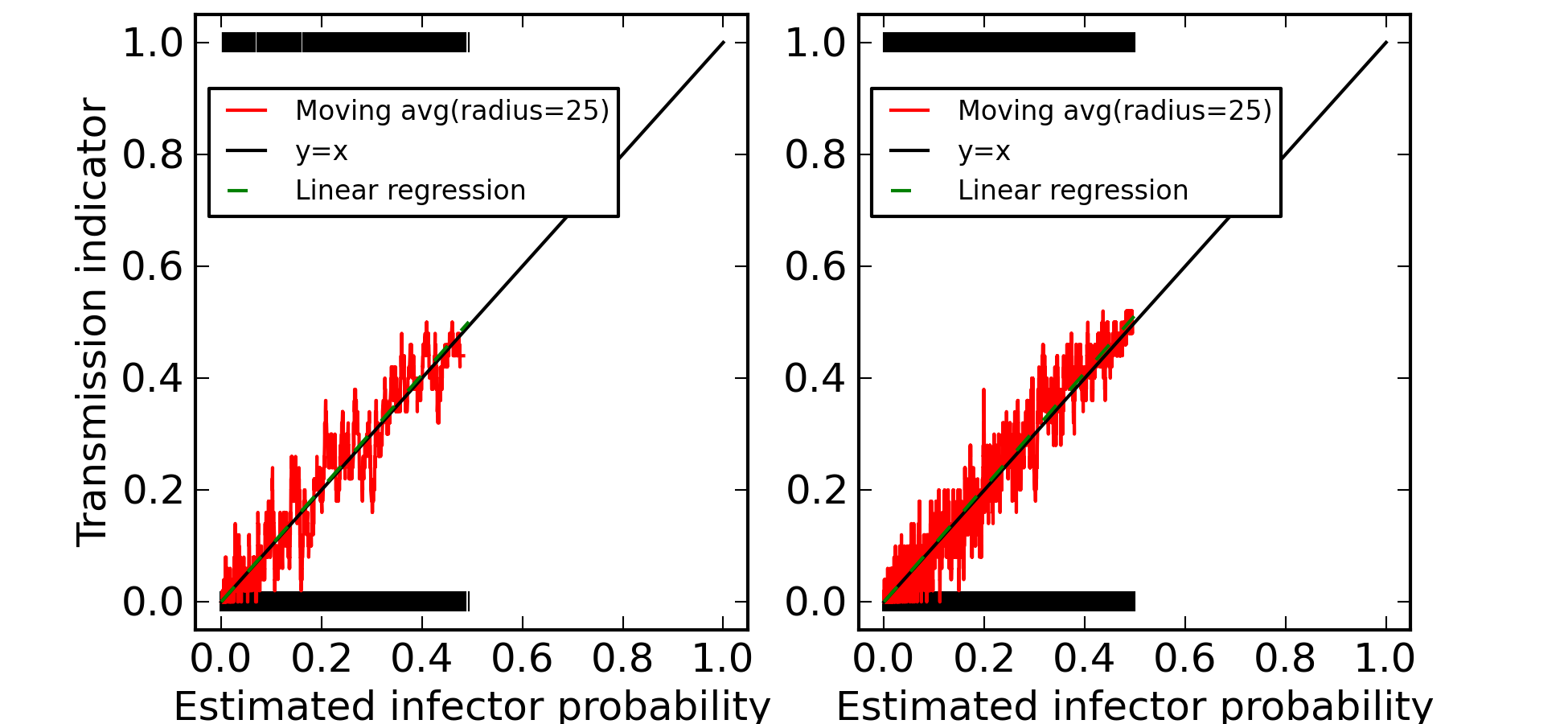

Supplement: Figure S2 — Regression of true transmission events (ticks on axis coded zero or one) on calculated infector probabilities . Left: samples of 10% at endemic equilibrium. Right: samples of 10% at peak prevalence. (PNG) [file pcbi.1003397.s002.png]

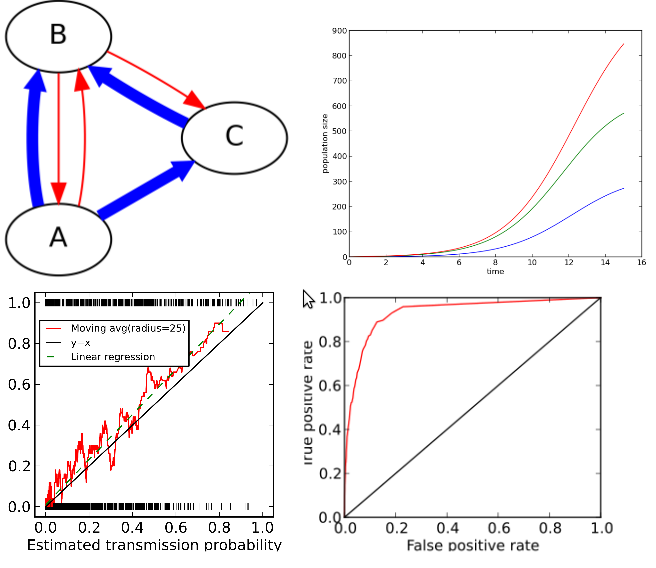

Supplement: Figure S3 — Top: Model structure and population size over time for a model with three states. Blue arrows represent birth within and between states. Red arrows represent migration between states. Bottom: Regression of true transmission events (ticks on axis coded zero or one) on calculated infector probabilities. At right is shown the ROC curve if infector probabilities are used for classification of the event that a putative transmission pair is real. (PNG) [file pcbi.1003397.s003.png]

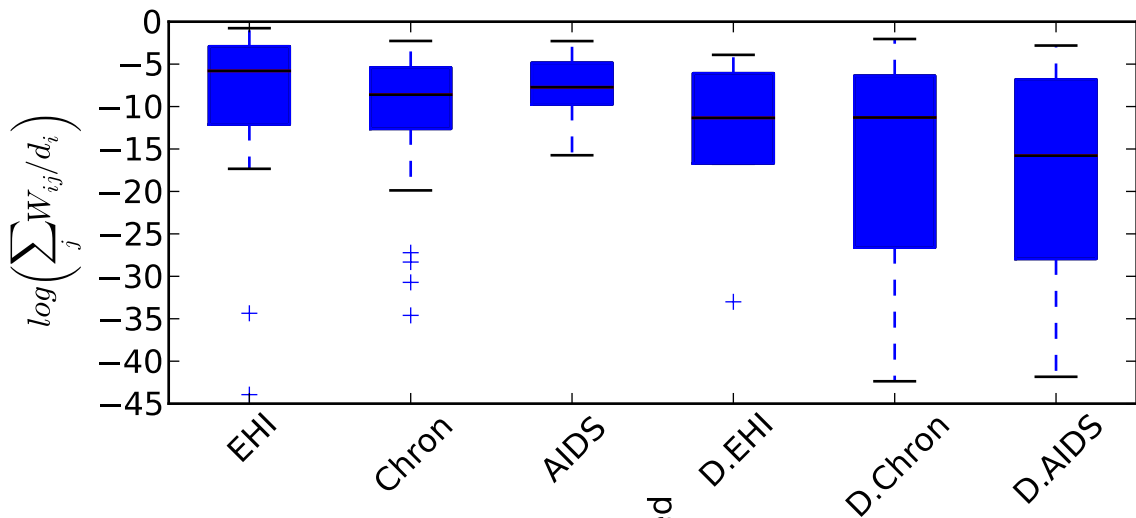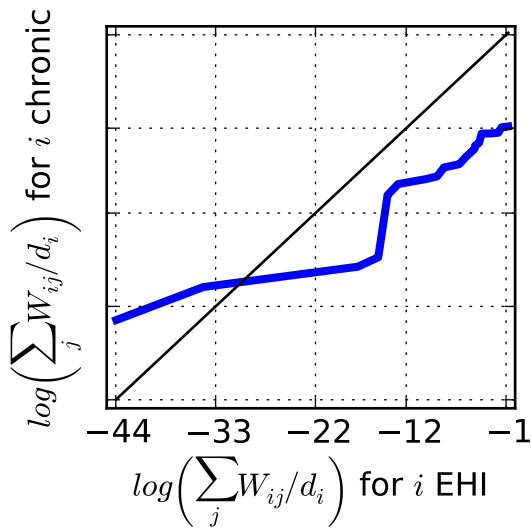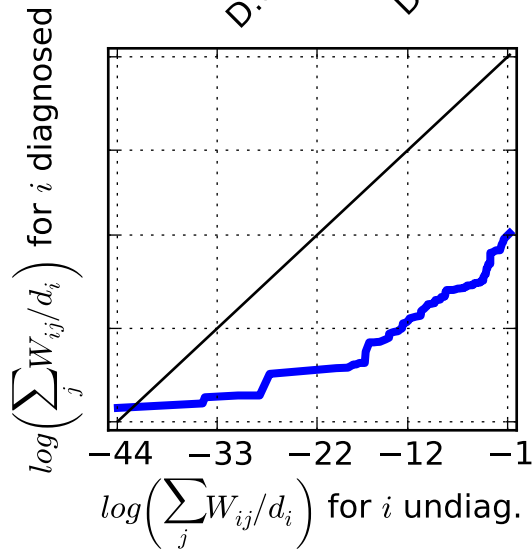

Supplement: Figure S4 — The log of the expected number of transmissions to at least one other sample unit is shown in aggregated form for different stages of infection and diagnosis status (top). Each stage is represented twice in this figure because an infected individual may be undiagnosed or diagnosed (labels prefixed with ‘D.’). A sampled lineage from an undiagnosed individual corresponds to a situation in which a pathogen is sequenced at the same time that the patient is diagnosed. A quantile-quantile comparison of the distributions of log infector probabilities for EHI and chronic stages is shown at bottom left. A quantile-quantile comparison for undiagnosed and diagnosed is shown at bottom right. (PDF) [file pcbi.1003397.s004.pdf]

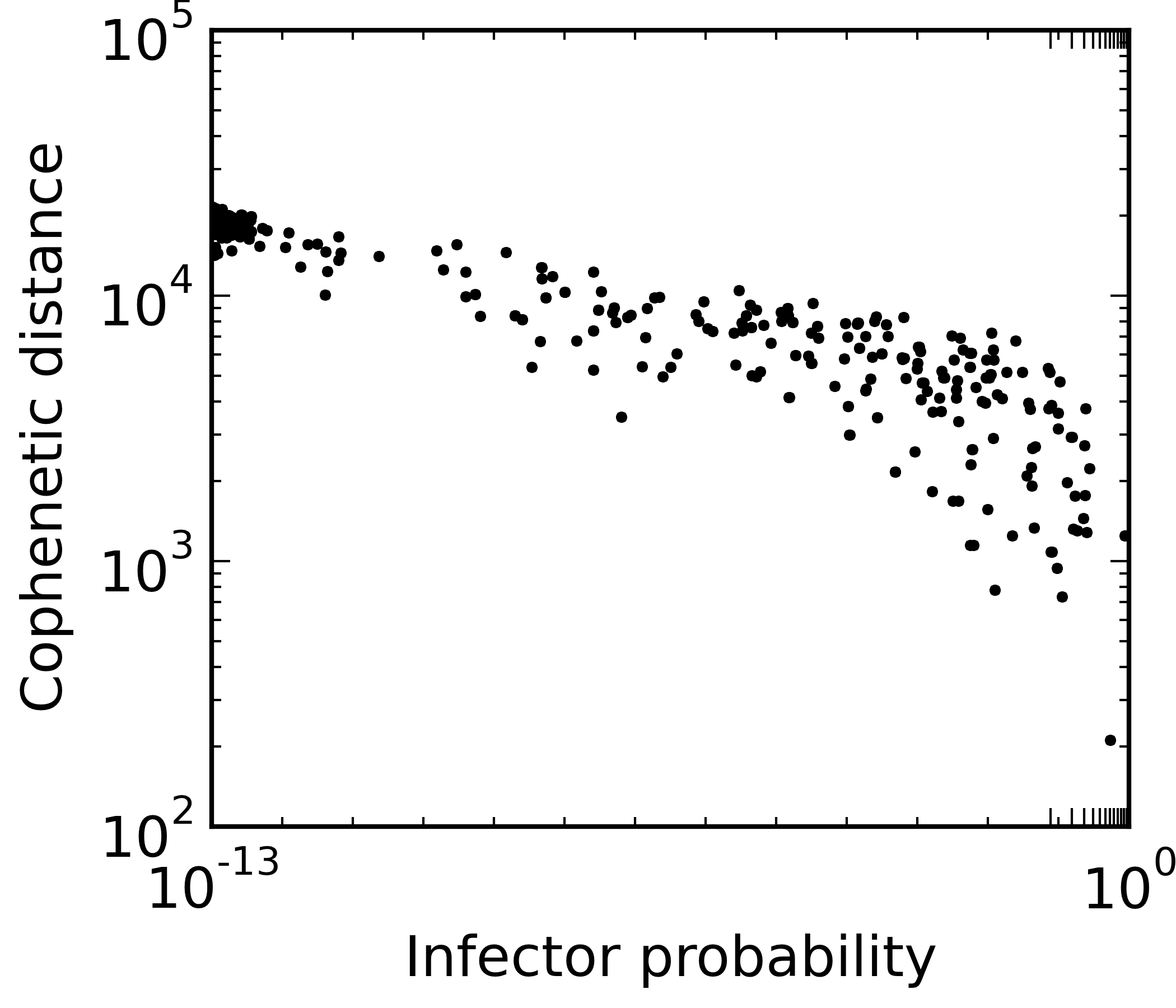

Supplement: Figure S5 — The cophenetic distance between each pair of tips in the HIV gene genealogy is shown versus the calculated infector probabilities. (PNG) [file pcbi.1003397.s005.png]
