# Supplementary material for: Inferring the Source of Transmission with Phylogenetic Data
Source: PLoS Comput Biol. 2013 Dec 19;9(12):e1003397. doi: 10.1371/journal.pcbi.1003397 (PMC3868546; doi:10.1371/journal.pcbi.1003397)
Supplement: Text S1 — Additional simulation experiments. (PDF) [file pcbi.1003397.s006.pdf]

Text S1 for *Inferring the source of transmission with phylogenetic data*

Erik M. Volz[1,*], Simon D.W. Frost[2]

1 Department of Infectious Disease Epidemiology, Imperial College London, UK

2 Department of Veterinary Medicine, University of Cambridge, UK

∗ **E-mail: e.volz@imperial.ac.uk**

A Additional simulations

To validate the numerical accuracy of our derivation of W, we have conducted many additional simulations under idealized situations with large sample sizes. Then, as in the main text, we compared estimated W to the frequency of true transmission events.

Estimated W_{ij} in samples from finite populations are subject to bias since the method makes approximations related to asymptotic behavior in the large population size limit. Nevertheless, we find this bias to be undetectable even in relatively small populations with 100-700 infected hosts.

S1 Experiment 1: SIRS at equilibrium and peak prevalence

The model is described by the following ODEs:

$$\begin{aligned} \dot{S} &= \mu R - \beta SI/N \\ \dot{I} &= \beta SI/N - \gamma I \\ \dot{R} &= \gamma I - \mu R \end{aligned} \tag{S1}$$

We used $\beta = 1.5, \mu = 0.1, \gamma = 1$. Discrete transmission and recovery events were simulated by setting rates to be equal to the solution of the deterministic model. Simulation trajectories are shown in figure S1.

A fraction $\phi = 0.1$ was sampled homochronously at two points in time: $t = 16.1$ which corresponds to peak prevalence and $t = 150$ which corresponds to endemic equilibrium. For each set of simulations we calculated:

- Number of pairs between sample units
- Number of true transmission events between sample units

- Expected number of transmissions based on estimated W. Summing across all simulations k and all pairs (i,j) in the k'th sample, this is

$$\sum_k \sum_{i,j \in \mathcal{S}(k)} W_{ij}^{(k)}$$

Results are shown in the following table:

Experiment	# simulations	# pairs	# transmissions	Exp. # transmissions
$\phi = 0.1$ at $t = 150$	527	13368	825	800
$\phi = 0.1$ at $t = 16.1$	631	45826	2343	2310

Define

$$A_{ij} = \begin{cases} 1, & \text{if i transmitted to j.} \\ 0, & \text{otherwise.} \end{cases}$$

In the absence of bias, the expected residual $E[W_{ij} - A_{ij}]$ should be zero where the expectation is taken across all pairs i, j in all simulations. A t-test was performed for H0: $E[W_{ij} - A_{ij}] = 0$. H0 was not rejected.

We also regressed A_{ij} on W_{ij}. Results are shown in figure S2. Results are also summarized in the following table:

Experiment	slope	intercept	s.e.	t-test p
$\phi = 0.1$ at $t = 150$	1.010	0.00	0.017	0.33
$\phi = 0.1$ at $t = 16.1$	1.027	-0.00	0.008	0.43

S2 Experiment 2: structure and large sample fraction

The demographic process was generated by the model generator available at *http://code.google.com/p/colgem/* and described previously in [1]. The model generator is a tool for generating demographic processes with births and migrations between discrete states. Population dynamics are described by a system of ODEs. The model had three states, a population size of 5000, and $n = 506$ individuals (approximately 30%) were sampled at uniform intervals from the last 5% of the population timeline. A discrete simulation was accomplished by simulating 5000 individuals with transmission and migration rates described by the solution of the deterministic model. Five simulations were carried out and results combined.

Figure S3 illustrates the model structure, population size over time, and infector probabilities. Unlike

in the HIV model, numerous transmission events are observed at higher infector probabilities because of the shorter generation times and higher sample fraction. The slope and intercept of the linear regression are respectively 1.11 and 0.01, reflecting a slight under-estimation of infector probabilities. In this case, infector probabilities are a highly accurate indication of whether transmission actually occured, and are useful for classification, as shown in the ROC curve of figure S3. AUC of ROC is 93%.

Supplementary References

1. Volz E, Ionides E, Romero Severson E, Brandt M, Mokotoff E, et al. (in press) HIV-1 transmission during early infection in men who have sex with men: A phylodynamic analysis. PLoS Medicine .
